# The Role of the Advanced Practitioner in a Comprehensive Lung Cancer Screening and Pulmonary Nodule Program

**DOI:** 10.6004/jadpro.2014.5.6.4

**Published:** 2014-11-01

**Authors:** Amanda E. Reid1, Lynn Tanoue2, Frank Detterbeck2, Gaetane Celine Michaud2, Ruth McCorkle3

**Affiliations:** 1University of Texas MD Anderson Cancer Center, Houston, Texas; 2Yale University School of Medicine, New Haven, Connecticut; 3Yale University School of Nursing, New Haven, Connecticut

The focus of many cancer centers has been primarily on the diagnosis and treatment of disease. While advancing treatments is important, prevention and early detection of disease needs to be formally integrated into comprehensive cancer cancers. It is the mission of the American Cancer Society to prevent and detect cancer early (American Cancer Society [ACS], 2008). Screening is the essential modality in achieving this mission.

It is estimated that more than half of all cancer cases worldwide can be prevented or detected early by screening (ACS, 2011). Effective screening tools reduce cancer mortality and influence cancer-specific survival rates. For decades, screening has been beneficial in early detection of breast, cervical, prostate, and colorectal cancers. Lung cancer, the leading cause of cancer death in both men and women, has not had the benefit of a screening modality; this is partially reflected in the poor 5-year survival rate of 16.6% (ACS, 2013; Howlader et al., 2013). When lung cancer is detected at an early stage and localized, the 5-year survival rate increases to 53.5% (Howlader et al., 2013). In 2011, the results of the National Lung Cancer Screening Trial (NLST) transformed the early detection of lung cancer with the demonstration of a mortality benefit related to screening with low-dose computed tomography (LDCT; Aberle et al., 2011).

Lung cancer screening is based on decades of clinical research. Prior randomized screening studies utilizing chest radiographs and sputum cytology proved insufficient to demonstrate a reduction in lung cancer mortality, the gold standard outcome of a screening tool (Oken et al., 2011; Marcus et al., 2000; Hocking et al., 2010). The NLST, a multi-institutional study sponsored by the National Cancer Institute (NCI), demonstrated for the first time that annual screening for 3 years with LDCT decreases the death rate from lung cancer by 20% (1 in 5 deaths). In this trial, LDCT scans were compared to chest x-rays as the screening intervention in a selected high-risk patient population. Individuals eligible for the study included those between the ages of 55 and 74 who had a 30–pack-year smoking history and who were either currently smoking or had quit within the prior 15 years.

The results of the NLST were pivotal in proving that screening for lung cancer can be beneficial. However, many unanswered questions about lung cancer screening still remain. Can the results of the NLST be generalized to individuals who had quit smoking more than 15 years ago? Can the results of the NLST be generalized to people who do not smoke but have other lung cancer risk factors? How can individuals who would most likely benefit from screening be reached in the community? Should primary care providers assume a central role in lung screening, knowing that the average nodule detection rate is 20% with greater than 90% of nodules detected being benign, which generates the frequent need for follow-up and further evaluation (Detterbeck, Mazzone, Naidich, & Bach, 2013)? Additionally, given that the NLST was conducted mainly at large academic NCI-designated medical centers, can screening be generalized to community practice without the support of a multidisciplinary team?

## SCREENING GUIDELINES

Since the publication of the NLST, many different organizations have released specific guidelines on how lung cancer screening should be implemented in clinical practice. The ACS, the American Lung Association (ALA), the American Society of Clinical Oncology (ASCO), the American College of Chest Physicians (ACCP), the National Comprehensive Cancer Network (NCCN), and the American Association of Thoracic Surgery (AATS) all recommend that screening should be performed within a multidisciplinary setting with a dedicated team of specialists who are experts in the field of lung cancer and are skilled in diagnosing, evaluating, and treating lung abnormalities (Wender et al., 2013; American Lung Association, 2012; Detterbeck et al., 2013; Bach et al., 2012; Wood et al., 2012; Jaklitsch et al., 2012).

The components of an ideal screening program have been outlined by Ahmad and Detterbeck (2012) as well as by Arenberg and Kazerooni (2012) and include patient risk assessment, education, counseling, risk modification (i.e., smoking cessation), appropriate patient selection, and standardized LDCT screening interpretation. Additionally, having a detailed and standardized process for nodule management, maintaining effective communication and follow-up of results, carrying out research to further refine the screening process (i.e., patient registry), and establishing and tracking quality metrics are all paramount. A dedicated program encompassing all of these elements assures that lung screening is executed according to evidence-based practice and that the appropriate individuals benefit from the screening process with minimization of harm and without excessive cost.

This article reports on the implementation and coordination of a comprehensive lung cancer screening program within an NCI-designated cancer center utilizing a multidisciplinary team approach, with a focus on the role of the advanced practitioner (AP).

## SCREENING PROGRAM SETTING

In 2010, Smilow Cancer Hospital at Yale–New Haven and Yale Cancer Center combined all cancer services and specialties as well as inpatient and outpatient in one facility. The transition into one facility allowed for a new philosophy of cancer care—one based on a single care delivery system involving 12 multidisciplinary specialty teams focused on achieving high-quality clinical outcomes and promoting clinical cancer research. In order to achieve this standard, APs were given the opportunity to develop their roles within the cancer specialty teams.

## DESIGN FRAMEWORK

**Figure 1:**
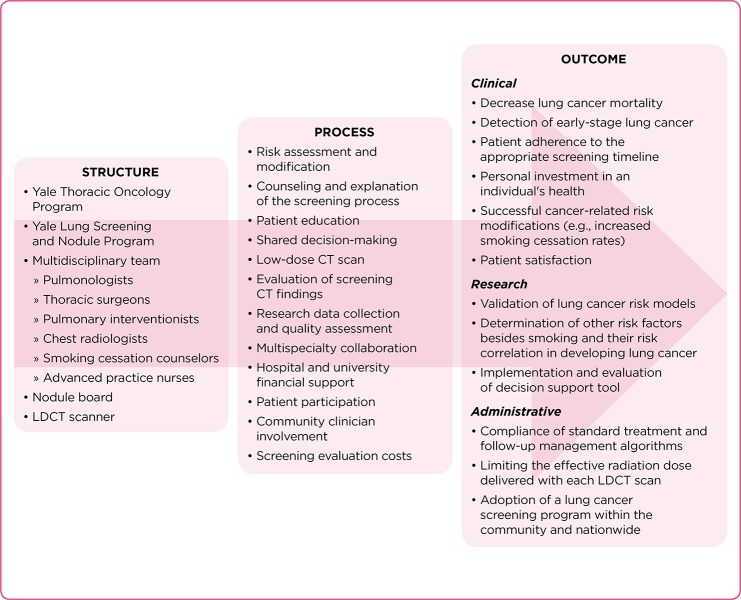
Yale Lung Screening and Nodule Program applied to Donabedian's Model of Quality Health Care. LDCT = low-dose computed tomography. Adapted from Donabedian (1980, 1988).

The framework used to design the disease teams’ delivery systems at Smilow Cancer Hospital was based on Donabedian’s Model of Quality Health Care (Figure 1; Donabedian, 1980). Donabedian identified three essential components (structure, process, and outcome) in establishing and assessing high-quality health service results. He described the *structural* measures of quality as the materials as well as the human and organizational resources that health-care professionals have at their disposal. He theorized that the distribution of these resources influences the types of care individuals are given. The measure of *process* represents the personnel who are delivering and receiving care (Donabedian, 1988). The involvement of patients in seeking and following up on the care that is given to them is vital, as is the providers’ involvement in evaluating, diagnosing, and treating the patients’ diseases. *Outcome* denotes the changes that occur in a patient’s physical, social, and psychological health status.

The coordination of care delivered on this platform of structure, process, and outcome ultimately impacts the successful or unsuccessful delivery of quality care. Donabedian’s framework served as a model in creating the Yale Lung Screening and Nodule Program (Yale Lung SCAN), a newly developed specialty within the Yale Thoracic Oncology Program, 1 of the 12 disease specialty teams.

## THE MULTIDISCIPLINARY TEAM

In order to establish a comprehensive lung cancer screening service, the physicians who established the Yale Lung SCAN acknowledged the need to recruit a multidisciplinary team of specialists including pulmonologists, chest radiologists, thoracic surgeons, thoracic oncologists, and smoking cessation counselors. They also recognized the value of integrating an AP into the team, understanding that APs meet patient needs across the continuum of care, from prevention and early detection of cancer to diagnosis and treatment, including guidance and counseling regarding lifestyle and risk modification (Oncology Nursing Society, 2007).

Studies have demonstrated that APs improve quality and continuity of care, enhance access to preventive services, and reduce health-care utilization and cost (Oliveria, Altman, Christos, & Halpern, 2002; Reed & Selleck, 1996). These findings are supported by the Robert Wood Johnson Foundation’s Initiative on the Future of Nursing at the Institute of Medicine (2010). Advanced practitioners are educated in health promotion, disease prevention, and risk reduction, which are all integral parts of cancer screening and early diagnosis of disease.

**Figure 2:**
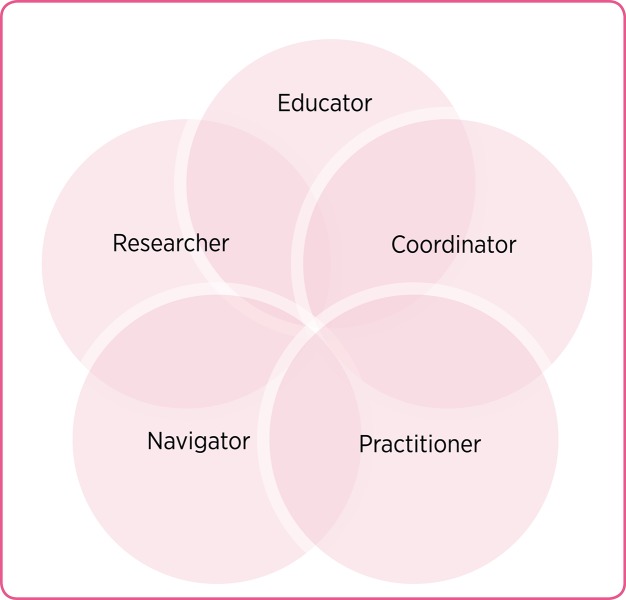
The multidimensional role of the advanced practitioner in the Donabedian Model of Quality Health Care.

The AP’s role in a dedicated lung cancer screening and pulmonary nodule program, such as Yale Lung SCAN, is multifaceted and distinct from the clinical focused role described by McCorkle et al. (2012), in which the majority of the time the AP is providing direct patient care in the inpatient and clinic-based setting. The AP is integrated throughout all aspects of the Donabedian Model of Quality Health Care, functioning as the program coordinator, system navigator, patient educator, research partner, and health practitioner (Figure 2). The Donabedian Model of Quality Health Care was selected because its components of structure, process, and outcome provide a framework for the AP to develop and implement a screening program.

**Coordinator**

Specific to the structure of the Donabedian model, the AP is a consistent member of the multidisciplinary team, having been charged with the development and coordination of the program. As the coordinator, the AP is intimately involved in organizing and managing the administrative needs of the program in conjunction with physician directors. Inherent in this process is the development and frequent updating of management algorithms based on current evidence; the development of education tools and sponsoring of events to enhance patient, community, and physician awareness of lung cancer screening; and the establishment of partnerships with clinicians in community practice to discuss ways in which program collaboration can occur. The AP is in charge of organizing weekly multidisciplinary pulmonary nodule board meetings, which are analogous to tumor boards, and linking pertinent clinical decision-making between providers and patients. Spearheading quality and safety initiatives, developing community outreach plans, and most importantly overseeing that the program operates seamlessly to best serve the needs of individuals enrolled in the program are all essential to the AP coordinator role.

**Navigator**

Navigation through a comprehensive screening program requires in-depth knowledge of the screening process and nodule management strategies to ensure that patients are guided through the initial evaluation and subsequent follow-up appropriately. The original purpose of the patient navigator role was to improve access to cancer screening (Freeman & Rodriguez, 2011). The AP in Yale Lung SCAN achieves this mission by educating at-risk individuals about the benefits of lung cancer screening and providing guidance and support to patients and their families throughout the screening continuum. Developing strategies to maximize compliance with recommended interventions is a vital part of this role.

The AP ensures that patients remain informed of the appropriate diagnostic evaluations and/or follow-up plan based on the findings of their initial and annual screening exams and ensures that "the loop is closed," including that all additional referrals (e.g., tobacco cessation), imaging studies, and procedures (e.g., bronchoscopy, CT-guided biopsy, surgery) are performed in a timely fashion. As a navigator, the AP also advocates for patient-shared decision-making and promotes ways in which physician recommendations and patient goals can be harmonized. Other cancer programs have demonstrated that the AP navigator increases patient satisfaction, facilitates access to timely care, and improves treatment outcomes (Rabinowitz, 2004; Seek & Hogle, 2007). Overall, the AP in the screening program allows the patient to receive maximum benefit from the screening process.

**Educator**

It is intrinsic to patient navigation that the AP serves as an educator. The AP informs patients and their families of the screening evaluative process, including the benefits and risks of screening, why lung cancer screening is different from other screening cancer modalities, and how patients can reduce their individual risk for developing lung cancer. Relaying the importance of reduction strategies such as smoking cessation and developing patient education materials is a key part of the AP’s role of clinical educator for patients and their families. Advanced practitioners are able to educate patients and their caregivers about potential recommendations, diagnostic procedures, and/or treatment modalities and what each recommendation means to their specific health situation. The AP in the screening program maximizes the many opportunities both in the tertiary and community settings to informally and formally educate other APs, physicians, nurses, and other staff members on the importance of discussing lung cancer screening with patients and referring them to a well-organized program.

**Researcher**

Since lung cancer screening is new to clinical practice, further research to refine the screening process is essential. Examples of clinical research intended to advance this area include the Yale Lung SCAN biorepository and decision support tool initiative. As a participating researcher in the screening program, the AP is responsible for identifying patients who are eligible to participate in active protocols. The AP supports the research mission of the lung cancer screening program by collaborating closely with the primary investigators and protocol personnel in obtaining consents, gathering clinical samples, and administering study questionnaires. The AP also captures and tracks the quality metrics that the program has underlined while measuring components and outcomes of a high-quality screening program (Table).

**Table 1:**
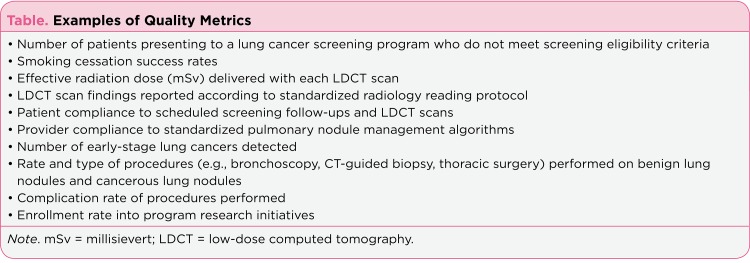
Examples of Quality Metrics

**Practitioner**

As a clinician, the AP assumes primary patient care responsibility for the individuals within the lung cancer screening and pulmonary nodule program. The AP performs a comprehensive cancer risk profile for each patient at the time of initial evaluation and subsequent follow-up, including utilization of validated risk-prediction models. The AP identifies and collaborates with interdisciplinary teams in the design of treatment plans. The AP monitors screening studies and other diagnostic imaging results, relays pertinent test information to patients and their families, and ensures seamless communication with the patients’ primary care providers.

The AP addresses any psychological burden, which frequently may surface during the screening process. Part of the AP responsibility is to help manage patient fears and anxiety and to recognize when the degree of these issues warrants formal psychological counseling. Current evidence on lung cancer screening and its influence on the quality of life and emotional state of an individual is limited. The majority of studies have demonstrated that patients experience a degree of short-term anxiety and distress from a range of 3 weeks to 6 months after an initial screening exam (van den Bergh et al., 2009; van den Bergh et al., 2008; Byrne, Weissfeld, & Roberts, 2008; Taylor, Shelby, Gelmann, & McGuire, 2004). Overall, the AP monitors all aspects of patient care across the continuum of the screening program.

## PROGRAM SUCCESS

Once the infrastructure of the program has been established, the highest priority is identifying and screening people who are at high risk for developing lung cancer. The program also must try to maintain individuals’ commitment to active long-term screening. The success of this phase is dependent on ongoing coordination with community providers. Once these steps are firmly established, the program will be positioned to participate in broader initiatives related to lung cancer screening, including the discussion of cost-effectiveness. Integrating the quality metrics captured will also give the program insight into how to adapt to the current and future patient population as well as the greater community.

## CONCLUSION

The role of the AP in a comprehensive lung cancer screening and pulmonary nodule program is multidimensional. Each function has unique characteristics that contribute to delivering evidence-based cancer screening care to individuals who are at risk for developing lung cancer, as well as to individuals who merit follow-up for screening-identified abnormalities. The AP contributes to the structure, process, and outcome of the program by serving as the program coordinator, navigator, educator, researcher, and practitioner and is essential in the program’s success in obtaining high-quality outcomes. In summary, the AP is the cornerstone of the multidisciplinary team, integrating patient-centered screening and nodule care into clinical practice.
